# Influence of bi- and tri-compartmental knee arthroplasty on the kinematics of the knee joint

**DOI:** 10.1186/1471-2474-12-29

**Published:** 2011-01-27

**Authors:** Markus Wünschel, JiaHsuan Lo, Torsten Dilger, Nikolaus Wülker, Otto Müller

**Affiliations:** 1 1Department of Orthopaedic Surgery, University Hospital Tübingen, Hoppe-Seyler-Str. 3, 72076 Tübingen, Germany

## Abstract

**Background:**

The cruciate ligaments are important stabilizers of the knee joint and determine joint kinematics in the natural knee and after cruciate retaining arthroplasty.

No in vitro data is available to biomechanically evaluate the ability of the anterior cruciate ligament (ACL) to maintain knee joint kinematics after bicruciate-retaining bi-compartmental knee arthroplasty (BKA).

Therefore, the objective of the current study was to investigate the kinematics of the natural knee joint, before and after installing bicruciate-retaining BKA and posterior cruciate retaining total knee arthroplasty. Specifically, we incorporated a dynamic knee simulator to simulate weight-bearing flexions on cadaveric knee specimen before and after surgical manipulations.

**Methods:**

In this cadaveric study we investigated rotational and translational tibiofemoral kinematics during simulated weight-bearing flexions of the intact knee, after bi-compartmental knee arthroplasty (BKA+), after resecting the ACL in BKA (BKA-), and after posterior cruciate retaining total knee arthroplasty (TKA).

**Results:**

Rotation of BKA+ is closest to the intact knee joint, whereas TKA shows significant differences from 30 to 90 degree of flexion. Within the tested flexion range (15 to 90 degree of flexion), there was no significant difference in the anterior-posterior translation among intact, BKA+, and TKA knees. Resecting the ACL in BKA leads to a significant anterior tibial translation.

**Conclusions:**

BKA with intact cruciate ligaments resembles rotation and translation of the natural knee during a simulated weight-bearing flexion. It is a suitable treatment option for medial and patellofemoral osteoarthritis with advantages in rotational characteristics compared to TKA.

## Background

Unicondylar knee Arthroplasty (UKA) is a well established treatment option for osteoarthritis of either medial or less often lateral knee joint compartment. Advantages include the minimal surgical exposure and the intact cruciate ligaments. It also provides the possibility to switch to a total knee arthroplasty (TKA) later, even though UKA conversion to TKA has been shown to be associated with poorer clinical outcome compared to primary TKA [1]. Shortcomings include: that it is unable to correct severe deviations of the mechanical axis; may result in restricted joint range of motion; requires intact ligamentous structures. UKA has been reported with excellent results comparable to those of TKA in longitudinal studies [2,3]. One of the main reasons for revision surgery after UKA is ongoing degeneration in the other compartments particularly in the patellofemoral compartment [4,5].

Bi-compartmental knee arthroplasty (BKA), replacing the medial and patellofemoral compartments, attempts to satisfy the fact that these compartments are most often affected by osteoarthritis [6,7]. The surgical approach for BKA is either to combine UKA and patellofemoral arthroplasty (PFA) in a modular design [8,9], or to use a recently developed non-modular femoral design [10-13]. In BKA, the anterior and posterior cruciate ligaments (PCL) can be preserved, and the reasons for retaining the cruciate ligaments in knee arthroplasty (KA) design include enhanced stability, decreased shear force between implant-bone interface, more physiological tibiofemoral kinematics, and maintenance of proprioception [14,15]. Therefore, theoretically, BKA may have same advantages of the UKA in terms of joint stability.

However, considering the articular surface in the tibiofemoral joint is altered after KA, the effect of the ACL on knee joint kinematics after bicruciate-retaining BKA may be different from that in the native knee. Although clinical data do exist [8,9,12], no in vitro data is available to biomechanically evaluate the ability of the ACL to maintain knee joint kinematics after bicruciate-retaining BKA.

Therefore, the objective of the current study was to investigate the kinematics of the natural knee joint, with bicruciate-retaining BKA and with posterior cruciate retaining TKA. Specifically, we incorporated a dynamic knee simulator to simulate weight-bearing flexions on cadaveric knee specimen before and after surgical manipulations.

We hypothesized that, (1) comparing to the native knee, the kinematics in internal-external rotation and anterior-posterior translation are not affected after a bicruciate-retaining BKA and (2) resecting the ACL in BKA as well as an ACL- sacrificing (PCL-retaining) TKA lead to different kinematic patterns.

## Methods

**Figure 1:**
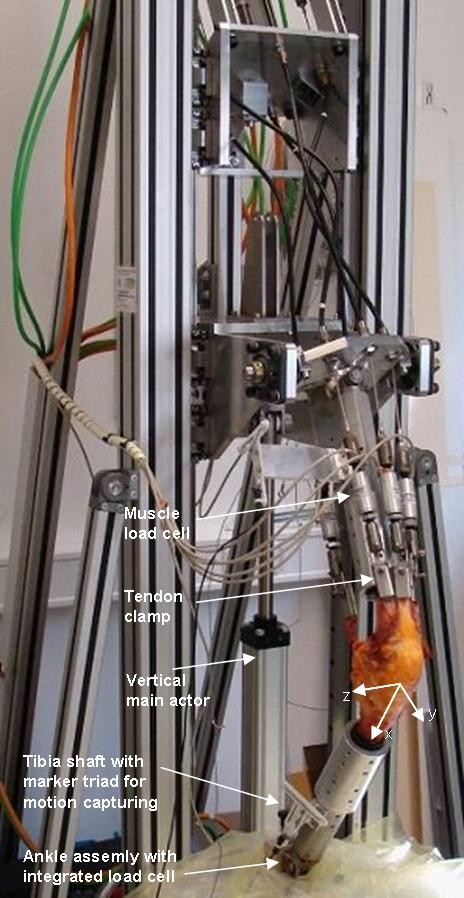
**Picture of the knee simulator**. The system is able to simulate a continuous weight-bearing knee flexion while moving the vertical frame and adjusting the muscle loads according to a predefined constant force measured by an uni-axial load cell in the ankle assembly simultaneously. Femoral and tibial shafts were secured into thick-walled aluminum adapter cylinders, and the adapter cylinders were attached to the fixation cylinders of the hip and angle assemblies via accurately-positioned screws. The kinematics of the knee joint was measured using ZEBRIS^® ^ultrasonic motion capture marker triads attaching on the tibial and femoral fixation tubes. Sketched arrows show the orientation of the coordinate systems in the knee joint

To simulate a dynamic weight-bearing knee motion, an upright knee simulator was used. This model has previously been described in detail [16]. The knee simulator consists of a vertical frame with a linear actuator (termed main actuator: linear electrical servo motor; Parker Hannifin, Offenburg, Germany), five smaller linear actuators (termed muscle actuators; Parker Hannifin, Offenburg, Germany) that generate the forces and motions of the five simulated muscles (Rectus femoris, vastus lateralis, vastus medialis, semitendinosus, and biceps femoris), a three degree-of-freedom (flexion-extension, adduction-abduction and vertical translation guided by a ball rail system) hip joint assembly, and a three degree-of-freedom (Internal-External rotation, flexion-extension, and adduction-abduction) ankle joint assembly (figure 1). With the designed degrees of freedom in the hip and ankle joints, the knee simulator allows unconstrained tibiofemoral movements in all six degrees of freedom [17]. Each of the six actuators can be controlled independently. The movement of the hip joint assembly is guided by a vertically-aligned ball rail bearing. In both of the hip and ankle assemblies, uni-axial (vertical) load sensors (Velomat, Kamenz, Germany) are mounted to measure the vertical reaction forces in the corresponding bearings (ankle and hip forces, respectively). Muscle forces were simulated by linear electrical servo motors via steel cables attached to the tendon of each muscle, and their forces were measured by uni-axial load cells.

Twelve fresh-frozen human cadaveric knee specimens with an age at the time of death of 75 ± 13 years (mean ± standard deviation) were studied. The femur and tibia were cut 15 cm from the joint line, and, while keeping the joint capsule and the collateral ligaments intact and the five aforementioned muscle tendons exposed, all other skin and soft tissues were removed. The fibula was secured to the tibia with cortical screws to prevent its motion during the test. Each of the femur and tibia was mounted onto a thick-walled steel cylinder using a bone cement compound (PMMA: Technovit 2060, Heraeus Kulzer, Hanau, Germany) and multiple accurately-positioned set screws. To achieve a secure grip of the tendon, a custom-manufactured metal tendon clamp was used to connect the tendon to the muscle actuator with a cable whose line of action is parallel to the femoral shaft.

To generate the weight-bearing knee flexions, the main actuator produced a continuous, descending motion of the hip assembly from 15 to 90 degrees of knee flexion with a constant rate of 1 deg/s. During this movement, the control system dynamically adjusted the muscle cable tension by varying their lengths so as to maintain a constant resulting ground reaction force on the ankle joint. The reaction force on the ankle joint, which holds equilibrium with the applied muscle forces quasi-statically, was assumed to characterize the amount of body weight. In order to prevent the tendon ruptures caused by excessive muscle forces [16], we selected a conservative ankle force of 50 Newton to simulate a portion of the body weight, which required the quadriceps-actuators to pull with a linearly rising force and a maximum of approximately 600N at 90 degrees of flexion. During knee flexion, the three quadriceps forces were always maintained identical to one another, while the hamstrings forces were kept constant at 10 N. A constant hamstring force was used to simplify the control algorithm and to reveal the effects of other factors of research interests. This knee flexion with a 50 N simulated partial body weight was repeated twice for each of the four different parameters.

To study the kinematics of the joint, the movements of the femur and the tibia were measured with a marker-based ultrasonic measuring system for 3D motion analysis (ZEBRIS^® ^CMS100, Isny, Germany) at a sampling rate of 1 Hz and spatial resolution < 1 mm [18]. A triad of ultrasound markers was attached at each of the femur and tibia fixation cylinders. To define the two body-fixed coordinate systems for the femur and tibia, we first recorded the positions of the medial and lateral prominences of the tibia plateau as reference points using a ZEBRIS^® ^stylus pointer when the knee was fully extended. Both of tibial and femoral coordinate systems were assumed to be identical at full extension, and their common origins at full extension were defined as the midpoint of the two digitized reference points. For each of the tibial and femoral coordinate systems, the flexion axis (z-axis) was defined along the line between the two reference points. The y-axis was defined as a vector normal to a plane constructed by the z-axis and the longitudinal axis of the respective segment shafts, which were recorded by the ultrasound sensor triads at the corresponding segments. The x-axis was then defined by the cross product of the y and z axes (figure 1). During the flexion/extension of the knee, the segment fixed coordinate systems at each instant were calculated according to the positions of tibial and femoral ZEBRIS^® ^marker triads. The tibial translation with respect to the femur was defined as the position difference between the centers of the two moving coordinates, and the relative orientation of the tibia with respect to the femur was calculated in terms of Euler angles (rotation sequence: flexion-extension, abduction-adduction, internal-external rotation).

**Figure 2:**
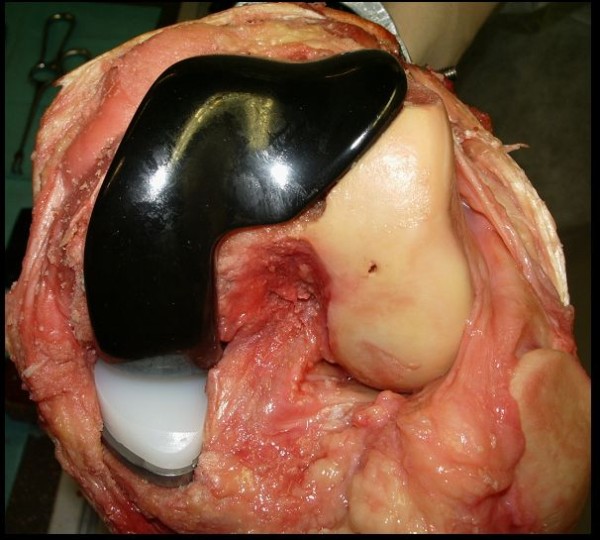
**Intraoperative situs after implanting the BKA**. Note the resurfaced medial and retropatellar compartment and the intact lateral compartment, patella as well as the cruciate ligaments. The little hole in the lateral condyle origins from a referencing-pin which was used to set referencing marks for the later switch to TKA.

To determine the effect of different KA-systems on the kinematics, the aforementioned protocol was performed on the intact knee specimen (termed "Intact" case). The same specimen was retested after a bicruciate-retaining BKA with intact and resected ACL (termed "BKA+"/"BKA-" case: JOURNEY DEUCE, Smith&Nephew, Memphis, Tennessee) and after ACL-sacrificing (PCL-retaining) TKA (termed "TKA" case: GENESIS II, Smith&Nephew, Memphis, Tennessee). The surgeries were performed by an experienced orthopaedic surgeon with the same surgical approach and instruments used for routine clinical care. In the BKA, a longitudinal incision was made over anterior aspect of the knee along the medial border of the patella. The bicruciate retaining prosthesis (figure 2) was then implanted using press-fit fixation. The femoral and tibial components were installed using standard intra- and extra-medullary alignment guides. Appropriate soft tissue balancing was performed in flexion and extension to achieve valgus-varus stability before closing the incision with sutures. After resecting the ACL through the partly reopened incision of the operated knee, a second KA with an ACL-sacrificing design was performed in the same knee specimen. The BKA was removed; tibia and femur were properly resurfaced and the ACL-sacrificing prosthesis was then installed. Soft tissue balancing was performed in flexion and extension to ensure valgus-varus stability before closing the incision.

To evaluate the effect of the KA design on the kinematics, at each of the tested knee flexion angles, we conducted a one-way repeated-measure analysis of variance (ANOVA) with knee states as the independent factors. A post hoc test using Tukey-Kramer method was also conducted to investigate the individual effect. A p-value less than 0.05 was considered to be statistically significant. The statistical analysis was conducted with a statistics computer software, SAS^® ^(SAS Institute Inc., Cary, NC).

The research presented in this work conforms to the Helsinki Declaration and to local legislation. It has been approved by the ethical committee of the medical faculty of the University of Tübingen.

## Results

**Figure 3:**
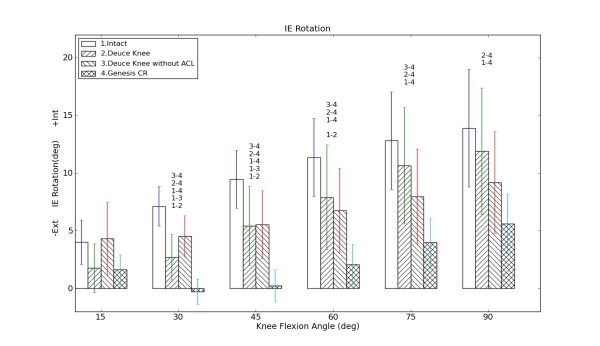
**Internal-External (IE) Rotation of the Tibia versus knee flexion angle**: Positive values represent internal tibial rotation; the lengths of the error bars indicate one standard error; the number-combinations denote significant differences in tibial rotation between the specified states (p < 0.05).

The rotational kinematics resulted from the four different knee states are shown in figure 3. The internal tibial rotation of the intact knee increased with increasing knee flexion, from 4 degree (15 degree of knee flexion) to 14 degree (90 degree of knee flexion). After installing BKA+, the internal tibial rotation reduced throughout the whole range of motion by up to 4 degree and was significantly different from the intact knee trials from 30 to 60 degree of flexion. After cutting the ACL (BKA-), the IE rotation did not show significant differences from the BKA+ cases.

After explanting BKA and performing TKA, we found significantly different rotational data between the intact knee and TKA trials from 30 to 90 degrees of flexion. The TKA design resulted in external tibial rotation from 15 to 30 degree of flexion and then changed into internal rotation from 30 to 90 degree of flexion. The rotation was approximately 8 and 5 degree less than those of the intact knee and BKA+ trials, respectively.

**Figure 4:**
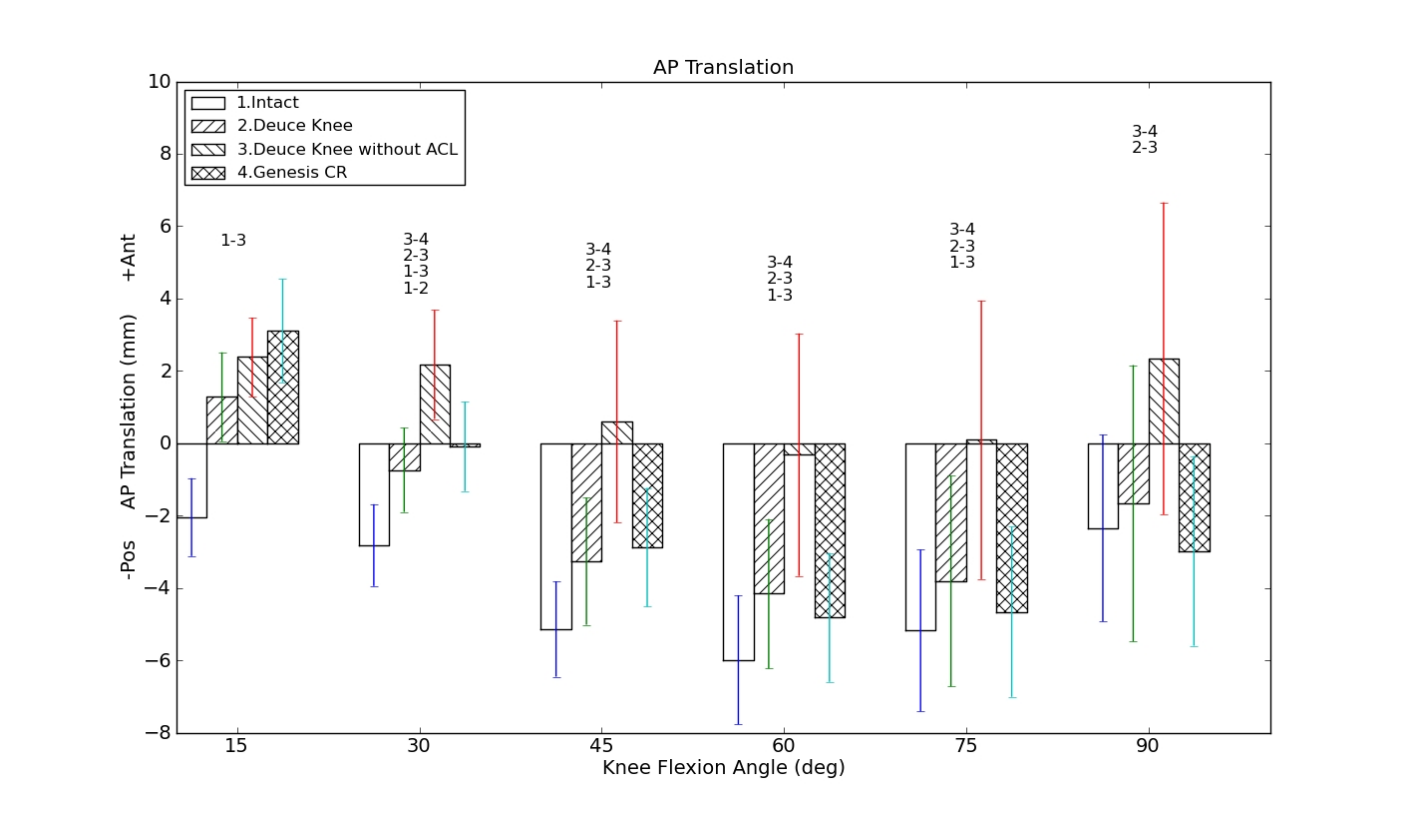
**Anterior-posterior (AP) Translation of the Tibia versus flexion angle**: Positive values represent anterior direction; the lengths of the error bars indicate one standard error; the number-combinations denote significant differences in tibial anterior translation between the specified states (p < 0.05).

The tibial translation data were presented in figure 4. Compared to the intact knee, all other cases (BKA+, BKA-, TKA) showed a greater anterior tibial translation from 15 to 75 degree of knee flexion. The anterior tibial translation resulting from BKA- trial was up to 6 mm greater than the intact knee trials. It was also the greatest among all four cases at almost all flexion angles (except 15 degree of flexion). The BKA+ and the TKA trials did not differ significantly from each other. No significant difference was found between the intact knee and TKA as well as between Intact and BKA+ (except at 30 degrees of flexion), accordingly.

## Discussion

In this study, we conducted simulated weight-bearing knee flexions to investigate the effect of bicruciate-retaining BKA (BKA+), ACL-resected BKA design (BKA-), and posterior-cruciate retaining TKA on translational and rotational knee joint kinematics. Since the investigations were performed with simulated muscle forces, the tibiofemoral kinematics of different KA designs with different ACL-states can be analyzed in a more physiologic fashion compared to previous studies [19]. The experimental design allows comparisons of multiple knee states on the same knee specimen and thus minimizes the effects of inter-specimen variations.

Concerning tibial rotation, TKA could not maintain physiologic kinematics from 30 to 90 degrees of flexion. Our results only show a slight internal rotation during flexion which was significantly less than that in the natural knee. This finding corroborates with other studies [20] and may result from the concave shape of the inlay, which limits rotational abilities. The amount of internal rotation in the intact knee in our setup was less than those reported in in-vivo MRI-studies [21]. It has been hypothesized [20] that the hamstring muscles have a significant influence on tibial rotation in vivo thus explaining lower degrees of rotation in vitro, where this muscle group usually is not addressed at all. We used a constant hamstring force of 10N bilaterally and did not apply any rotational torque. Further investigation on this topic is clearly warranted.

The BKA+ and BKA- trials did not show a significant difference in tibial rotation, which leads to the assumption that the ACL does not have an influence on tibial rotation during a simulated weight-bearing flexion. This is in contrast to several other studies [19,22] and might be caused by high quadriceps-forces used in this study to simulate partial body weight. Another difference is that we consistently increased quadriceps-force during flexion as it is in vivo, taking into account the changing lever-arms during flexion, while other studies usually apply the same amount of force to the quadriceps-muscle thus producing a less physiological condition [20,23].

Figure 3 shows that the intact knee and BKA+ have almost parallel rotational curves. The fact that BKA+ rotates less may be explained by the influence of the surgery leading to changes of the soft tissue.

Our findings show that bicruciate-retaining BKA as well as TKA preserve the natural-knee anterior-posterior translation with a minimally more anteriorly positioned tibia. This may result from a slight ligamentous laxity caused by the surgical procedure. This is in contrast to the findings in previous in vivo clinical investigations [24] that the patients with the ACL-retaining TKA had more natural tibiofemoral kinematics than those with PCL-retaining TKA did during treadmill gait and stair stepping.

Our findings also suggest the geometrical congruity of the TKA used in this study was able to compensate for the missing ACL. However, after cutting the ACL in BKA (BKA-), the tibia significantly moved anterior compared to all other states. This is because the tibial plateau in BKA is flat and thus not able to compensate for the missing ACL. The designs of the implanted femoral components in TKA and BKA (except for the missing lateral condyle in BKA) are alike and thus do not cause differences in kinematics. Other studies also emphasize the importance of the articular geometry after KA [25].

Several limitations exist in the current study. First of all, we simulated only a partial body weight. This was because the quadriceps tendons in some of the specimens could not sustain the corresponding muscle forces resulting from higher body weight simulation. In order to avoid the risks of tendon rupture under high simulated muscle loading, we compromised to simulate only a portion of the body weight [20]. Moreover, previous investigation [16] has shown that the change in the knee kinematics profile is not sensitive to the increasing simulated body weight. Therefore, we believe that qualitative clinical insights can still be elucidated with the partially loaded knee. However, although the results are in concordance with in vivo studies [26-28] they can not definitely be extrapolated to predict the knee kinematics under full body weight in vivo. A second limitation is that the tested specimens did not display the indications, such as medial compartment degeneration and ligamentous/capsular changes typical in patients eligible for KA. Therefore, extra care was taken by the surgeon to ensure that all the ligaments were properly tensioned and knee axes were well aligned after implantation of the components. A third limitation in this study is that the knee flexion only started from 15 degree, because the joint could not be flexed from fully-extended position by only applying a partial body weight and quadriceps force. This is due to the setup of the knee kinemator control: Since we drive the kinemator fully force-controlled the specimen might hyperextend and thus be destroyed if the extension angles reach below 15 degree. The information within first 15 degree of flexion is thus missing. A fourth limitation is the hamstring forces were kept constant during the simulated flexion. This is mainly because the multiple agonist and antagonist muscle forces composed a mechanically-indeterminate system, and it is not likely to determine their individual contributions. We therefore believe the use of constant hamstring forces can isolate the effects of other factors without sacrificing the fidelity of the model.

## Conclusion

Our data show that the translational and rotational knee joint kinematics after bicruciate-retaining BKA resembles that of the native knee. On the other hand PCL-retaining TKA results in less rotation and similar translation during a partially weight-bearing flexion. Our findings suggests that, provided functional ligamentous structures, bicruciate-retaining BKA is a suitable treatment option for medial and patellofemoral osteoarthritis of the knee joint with advantages in rotational characteristics compared to TKA.

## Competing interests

The presented study was partly funded by Smith&Nephew.

## Authors' contributions

MW conceived the study, performed the study including surgery and drafted the manuscript. JL participated in the design of the study and performed the statistical analysis. TD assisted and coordinated the study. NW participated in the design and coordination of the study. OM operated the knee simulator and performed the statistical analysis. All authors read and approved the final manuscript.

## Pre-publication history

The pre-publication history for this paper can be accessed here:

http://www.biomedcentral.com/1471-2474/12/29/prepub
